# Persistent Unilateral Sore Throat: Should It Be Included in the 2-Week Wait Referral Criteria by NICE

**DOI:** 10.1155/2019/4920514

**Published:** 2019-05-05

**Authors:** Ahmed Allam, Hazem Nijim

**Affiliations:** ^1^Otolaryngology Department, Sheffield Teaching Hospitals NHS Trust, UK; ^2^Department of Otolaryngology, Faculty of Medicine, Mansoura University, Egypt; ^3^Otolaryngology Department, Derby Teaching Hospitals, UK

## Abstract

**Design and Setting:**

A retrospective study was conducted on all 2WW referrals made to our tertiary head and neck centre in a 12-month period.

**Methods:**

Sensitivity, specificity, and PPV of presenting complaints in H&N cancer diagnosis using Excel® and the statistical package SPSS®.

**Results:**

The sensitivity and specificity of 2005 NICE guidelines in detecting H&N cancers were 91.2% and 59%, respectively; their PPV was 9%. The sensitivity and specificity of 2015 NICE guidelines were 75.4% and 71%, respectively; their PPV was 10.3%. Eight out of 85 patients who presented with unilateral sore throat for more than 4 weeks, with or without otalgia and normal otoscopy, had H&N cancer (PPV 9.5%).

**Conclusions:**

Although the 2015 NICE guidelines have a high rate in detecting H&N cancers, consideration of reincluding unilateral sore throat in the referral criteria might be necessary.

## 1. Introduction

Following the 1998 White Paper, a new approach was established by Cancer Services Collaborative to facilitate quick review for patients with a suspected cancer [1]. In 2000, the Department of Health (DoH) introduced the guidelines and established 2 week wait (2WW) urgent referral for patients with symptoms suspicious of cancer in order to have an early diagnosis, treatment, and subsequently better prognosis for cancer patients in UK.

Regarding head and neck (H&N) cancer, the DoH sets ten symptoms with high possibility of cancer for H&N referrals in 2000. These symptoms were refined to 8 symptoms by the National Institute for Health and Care Excellence (NICE) in 2005 [2]. In 2015, NICE published new guidance taking into consideration 3% positive predictive value (PPV), a threshold value to support the recommendations for pathway of suspected cancer. It refined symptoms to subsites with 5 symptoms suspecting head and neck cancer (HNC) for the urgent referral from general practice to secondary care [3] (Table 1).

Many studies have assessed the efficacy of 2WW pathway in the detection of cancer. Kumar et al. 2012 found that the cancer detection rate based on the 2000 NICE guidelines ranged from 6.3% to 14.6% [4]. Another study conducted on 4123 2WW referred patients found that 6% of them had H&N cancer diagnosis [5].

In our study, we focus on the efficacy of NICE guidelines in H&N cancer detection rate, compare the 2015 and 2005 NICE in detecting H&N cancer, and evaluate the positive predictive value (PPV) of persistent unilateral sore throat more than 4 weeks with or without otalgia, a symptom that was not included in the 2015 NICE guidelines.

## 2. Materials and Methods

We retrospectively studied all 2WW referrals seen in our H&N tertiary centre in a 12-month period (January 2016 to December 2016). Data was collected from hospital electronic database and patients' medical notes. We excluded patients with thyroid cancer, lymphoma, previous H&N cancers; those who did not attend their appointments or where data was missing and those cases managed by our oral and maxillofacial colleagues.

The collected data were analysed using Excel® (Microsoft, Redmond, WA, US) and the statistical package SPSS®. 2010 and 2015 NICE Guidance's sensitivity, specificity, and positive predictive value (PPV) were calculated.

## 3. Results

### 3.1. 2-Week Wait Referrals

A total number of 1553 patients were referred from primary care general practitioners (GP) through 2WW pathway to our H&N centre within a 12-month period. We excluded 186 patients who did not fulfill inclusion criteria. Out of the remaining 1367 patients, 627 were males (45.8%), and 740 were females (54.2%). Their mean age was 59 (age range 16-95). 82.3% were above 45 years of age.

### 3.2. Cancer Detection Rates through the 2WW Pathway

Only 57 out of the 1367 patients (4.2%) we included in this study were diagnosed with H&N cancers. The cancer detection rate of the 2005 guidelines was 9% (52/577) and the detection rate of the 2015 guidelines was 10% (42/417).

The commonest symptoms referred via the 2WW pathway and those with high PPV for detection of cancer are presented in Tables 2 and 3. For example, we have the following:,

1. *Those patients presented with a new or persisting neck lump for > 3 weeks:* 32 out of 270 patients, who were referred with this symptom, were diagnosed with cancer (PPV 12.2%).
2. *Hoarseness of voice persisting more than three weeks:* 10 out of 147 patients who were referred with this symptom were diagnosed with cancer (PPV 6.8%).
3. *Persistent sore throat:* 9 out of 137 patients who were referred with this symptom were diagnosed with cancer (PPV 6.5%).

### 3.3. Efficacy of 2005 and 2015 NICE Guidelines

The sensitivity and specificity of the 2005 NICE guidelines were found to be 91.2% and 59%, respectively, in detecting H&N cancers via the 2WW pathway. Their PPV was 9%. The sensitivity and specificity of the 2015 NICE guidelines were found to 75.4% and 71%, respectively. Their PPV was 10.3%.

### 3.4. Noncancer Diagnoses following a 2WW Referral

The most common noncancer diagnosis in our 2WW referrals was laryngopharyngeal reflux (454 patients). No abnormality was found in 241 patients. Other diagnoses can be seen in Table 4.

### 3.5. Analysis of Persistent Sore Throat

We received 137 referrals of patients with a persistent sore throat. 9 of them were found to have H&N cancer. In further analysis, we found the following (Table 5):

(i) 17 (12.4%) patients had persistent unilateral sore throat for less than four weeks: none of them was diagnosed with H&N cancer.

(ii) 57(41.6%) patients had persistent unilateral sore throat more than four weeks. Cancer was detected in 5 patients (PPV 8.8%).

(iii) 28 (20.4%) patients had persistent unilateral sore throat with otalgia with normal otoscopy. Cancer was detected in 3 patients (PPV 10.5%).

(iv) The remaining 35 patients had a nonspecific persistent sore throat. One patient was cancer positive (PPV 2%).

*Overall, unilateral sore throat for more than 4 weeks with or without otalgia and normal otoscopy has a high PPV or 9.5*%.

## 4. Discussion

### 4.1. Comparison with Existing Literature

#### 4.1.1. Cancer Detection Rates

2015 NICE Guidelines had lower sensitivity (75.4%) in comparison with 2005 guidelines (91%); however, 2015 guidance has higher PPV for diagnosing cancer and higher specificity.

#### 4.1.2. Persistent Unilateral Pain in Head and Neck

Persistent unilateral sore throat with otalgia is an important presenting complaint for diagnosing H&N cancer, especially for oropharyngeal, laryngeal, and hypopharyngeal malignancy [6].

In our study analysis, unilateral sore throat for more than 4 weeks with or without otalgia had a 9.5% PPV in H&N cancer diagnosis. Tikka et al. [7] showed higher PPV (14.2%) for unilateral persistent neck pain more than 4 weeks with or without otalgia and normal otoscopy, but this finding was based on a smaller number of patients (49). This symptom of unilateral sore throat was not included in 2015 NICE guidelines for 2WW referral criteria. Although there has been no official reason for that, we assume that general practice is overwhelmed with a wide variety of sore throat presentations. Therefore onward referral to a tertiary centre might not necessarily be the best option in all these cases. Our study suggests that unilateral sore throat for more than 4 weeks has a high PPV for diagnosing cancer and we would recommend that this symptom is added on the following NICE guidelines on 2WW for H&N cancers.

### 4.2. Strengths and Limitations

The major drawback of our study is that it is a retrospective study. Most published studies on 2WW referral pathways were retrospective; a prospective design would offer more robust outcomes. Data were recorded in the patient first appointment by different grades of ENT doctors. This could be considered bias in methodology. This study is unique as it has included the highest number of referred patients in a single centre, since the 2015 NICE guidelines were published.

This was a retrospective cohort and the population of this study was limited to the patients who were only referred through 2WW pathway as a 2ww to our Head and Neck Unit at Sheffield Teaching Hospitals, Sheffield.

Unfortunately, we do not have data about the total numbers of all patients with sore throat admitted to our ENT not as a 2WW. This was out of the study objectives. I think it would be a great idea to perform a study looking at that but that would mean lots of challenges (we see more than 25.000 new referrals to ENT at Sheffield Teaching Hospitals per year)

We found that the majority of these patient were referred as a 2WW from primary care after exhausting all treatment medications (various lots of antibiotics and pain killers) and patients are still symptomatic; however, we could not analyse it as it was missed in some patients, a lot depending on how well the patients were interviewed regards to symptoms and previous treatments received.

Other factors which could have triggered the 2ww referral were smoking and alcohol history.

Head and neck cancer symptoms are unspecific; oropharyngeal cancers can be diagnosed in patients presenting only with sensation of lump with no other ENT symptoms.

However, from our study we would recommend our colleagues in primary care to have a very low threshold to refer a patient as a 2WW for being suspicious of head and neck cancer especially if they have unilateral sore throat for > 5 weeks and especially if that has become associated with otalgia with normal otoscopy.

Our colleagues in primary care face many challenging in screening head and neck cancer patients taking in consideration that many symptoms are unspecific and they have very limited time for patients which could make taking a comprehensive clinical history very challenging.

### 4.3. Implications for Research and/or Practice

2WW referrals have a high detection rate of cancers if they are compliant with 2015 NICE guidelines (10%). We recommend investing more resources in more education in H&N cancer symptoms for general practitioners. This can be achieved with the help of an ‘e-Learning for Healthcare' module online, as well as continuous feedback from H&N colleagues (via written feedback, meetings, and telephone advice). The aim would be to be confident in making (or ruling-out) a cancer diagnosis have more appropriate 2WW referrals [8].

Open discussion between GPs and patients around cancer should be encouraged when referring them for symptoms suspicious of cancer in order to line up referrals with NICE guidelines and to decrease patients pressure on primary practice for urgent referrals [9].

## 5. Conclusion

The newest NICE guidelines for an urgent referral via the 2WW pathway in 2015 have higher specificity and positive predictive value for cancers in comparison to previous guidelines, possibly because it narrowed the referral items to only 5 symptoms. Our study confirmed the low cancer detection rate via the 2WW urgent referral pathway, similar to previous studies [7, 10, 11].

Persistent unilateral sore throat for more than 4 weeks with or without otalgia with normal otoscopy was included in the 2005 NICE guidelines for an urgent referral via the 2WW pathway but not in the 2015 NICE updates with no cited reasons [7]. This study raises our concern that this symptom has a high PPV (9.5%) of positive cancer and should be reconsidered when designing future guidelines.

## 6. Summary

- The newest NICE guidelines for an urgent referral via the 2WW pathway in 2015 have higher specificity and positive predictive value for cancers in comparison to previous guidelines.
- Persistent unilateral sore throat for more than 4 weeks with or without otalgia with normal otoscopy has high cancer predictive value and was not included in NICE guidelines 2015.
- Persistent sore throat can be reconsidered to be included in the 2-week wait referral criteria updates by NICE.

## Figures and Tables

**Table 1:** Symptoms suspecting cancer for urgent referral.

DoH 2000 Guidelines	NICE Guidelines 2005	NICE Guidelines 2015
Ulceration of oral mucosa persisting for > 3 weeks	Ulceration of oral mucosa persisting for > 3 weeks	Ulceration of oral mucosa persisting for > 3 weeks

All red or red and white patches of the oral mucosa	Unexplained red and white patches (including suspected lichen planus) of the oral mucosa	A red or red and white patch in the oral cavity consistent with erythroplakia or erythroleukoplakia

Hoarseness persisting for >6 weeks	Hoarseness persists for more than 3 weeks	Aged 45 or above persistent unexplained hoarseness

Unresolving neck masses for > 3 weeks	Unresolving neck lump for > 3 weeks	Persistent unexplained lump in the neck

Unexplained tooth mobility not associated with periodontal disease	Unexplained tooth mobility not associated with periodontal disease	Lump in the lip or oral cavity

Unilateral nasal obstruction particularly when associated with purulent discharge	Unexplained persistent sore or painful throat	

Cranial neuropathies	Persistent swelling in the parotid or submandibular gland	

Orbital masses	Unilateral unexplained pain in the head and neck area for more than 4 weeks, associated with otalgia but with normal otoscopy	

Dysphagia persisting for 3 weeks		

Oral swellings persisting for > 3 weeks		

**Table 2:** Cancer detection rate in patients referred through 2 ww pathway and compliant with NICE guidelines.

Symptoms	Frequency (%)		Cancer positive	Included in 2005 NICE guidelines	Included in 2015 NICE guidelines	PPV (%)
Neck lump: new or persisting for > 3 weeks	270	19.8%	32	Yes	Yes	12.2%

Hoarseness Persisting for > 3 weeks	147	11.1%	10	Yes	Yes	6.8%

Persistent painful sore throat	137	10%	9	Yes	No	6.5%

Unilateral neck pain and otalgia with normal otoscopy	23	1.3%	1	Yes	No	4.3%

Total	577		52			9%

**Table 3:** Cancer detection rate in a patient referred through 2 ww pathway and NOT compliant with NICE guidelines.

Symptoms	Frequency (%)		Cancer positive	Included in 2005 NICE guidelines	Included in 2015 NICE guidelines	PPV (%)
The sensation of a lump in the throat	181	14.3%	0	No	No	0%

Intermittent hoarseness	115	9.1%	0	No	No	0%

Intermittent sore throat	78	5.7%	0	No	No	0%

Discomfort in throat	78	5.7%	0	No	No	0%

*Unilateral nasal obstruction and discharge *	*53*	*3.9*%	*1*	*No*	*No *	*1.89*%

Unilateral OME	42	3.1%	0	No	No	0%

*Dysphagia for > 3 weeks *	*33*	*2.4*%	*2*	*No*	*No *	*6.06*%

Intermittent sore throat and hoarseness	33	2.4%	0	No	No	0%

Unilateral nasal obstruction only	27	2.1%	0	No	No	0%

*Presence of blood in the mouth*	*24*	*1*%	*1*	*No*	*No *	*4.17*%

Unilateral nasal discharge only	22	1.6%	0	No	No	0%

Unexplained septum ulcer	18	1.3%	0	No	No	0%

*Persistent unexplained facial palsy *	*18*	*1.2*%	*1*	*No*	*No *	*5.56*%

Unilateral neck pain	16	1.2%	0	No	No	0%

Tightness throat	12	0.9%	0	No	No	0%

Smell disturbance	11	0.8%	0	No	No	0%

Unexplained septum Perforation	9	0.7%	0	No	No	0%

Persistent unexplained sore nose	9	0.7%	0	No	No	0%

Taste disturbance	6	0.4%	0	No	No	0%

Blood in the ear	5	0.4%	0	No	No	0%

Total	*790*		*5*			

**Table 4:** Final diagnosis of patients referred through 2 ww pathway.

Diagnosis	Frequency	Percent
Head and neck malignancy	*57*	*4.2*%
Laryngopharyngeal Reflux	454	33.2%
No abnormality /reassured	240	17.6%
Functional dysphonia	96	7%
Globus pharyngus	76	5.6%
Reactive /Normal lymph nodes	57	2.8%
Benign salivary glands lesions	38	2.8%
Idiopathic unilateral vocal cord Palsy	36	2.6%
Reinke's oedema	25	1.8%
Infections	23	1.7%
Vocal cord nodules	19	1.4%
Septal deviation	17	1.3%
Cervical lipoma	16	1.2%
Laryngeal polyps	12	0.9%
Sebaceous Cysts	12	0.9%
Retention tonsillar cysts	10	0.8%
Cervical TB	7	0.5%
Hyperkeratosis vocal cord	7	0.5%
Laryngeal papillomatosis	5	0.4%
Mild cord dysplasia	5	0.4%
Moderate vocal cord dysplasia	3	0.2%
Cervical toxoplasmosis	2	0.1%
Bronchogenic carcinoma	*2*	*0.1*%
Granuloma arytenoid	2	0.1%
Vallecular cysts	2	0.1%
Neurofibroma	2	0.1%
Carotid body tumour	2	0.1%
Thyroglossal cyst	2	0.1%
Other diagnosis	138	10.1%

Total	1367	100%

**Table 5:** Analysis of a persistent sore throat.

Symptom	Frequency (%)		Cancer positive	
Unilateral pain in throat < 4 weeks	17	12.4%	0	0%
Unilateral pain in throat >4 weeks	57	41.6%	5	8.8%
Unilateral pain in throat and otalgia with normal otoscopy	28	20.4%	3	10.7%
Nonspecific Sore throat	35	25.5%	1	2%

Total	137	100%	9	6.5%
Unilateral pain in the throat more than 4 weeks with or without otalgia with normal otoscopy	85 /137	62%	8	9.5%

## Data Availability

The data used to support the findings of this study are available from the corresponding author upon request, and the patient's data used to support these findings are included in the article.
